# Book Reviews

**Published:** 1899-06

**Authors:** 


### BOOK REVIEWS, PAMPHLETS, EXCHANGES.

### BOOK REVIEWS.

[Contributions solicited for review.]

THE INTERNATIONAL MEDICAL ANNUAL AND PRACTITIONERS' INDEX. Published by E. B. Treat & Co., New York. Price $3.00.

This is the Medical Annual for 1899, making the seventeenth year of its publication. Like all of its predecessors it is a volume containing a great deal of ready reference information of things medical and surgical which have occurred during the last twelve months. It is by no means complete yet; much of the matter selected by each contributor is of material value. A great many important advances are not mentioned under the various headings. For instance, a great deal has been written during the last year on *Cataract,* and yet, the only thing found under that head is a review of an article by Von Rothmund of Munich, who reports 400 extractions of senile cataract. We would certainly expect to find some mention made of the after treatment of cataract without bandage as practiced by Hjorta. Under many subjects we can find that many important things have been omitted. However, the work is fully up to the standard of its predecessors, and contains much information that is valuable. Especially valuable is the latter portion of the book, which treats of "Notes on Legal Decisions."

ANNUAL AND ANALYTICAL CYCLOPEDIA OF PRACTICAL MEDICINE. By Charles E. de M. Sajous, M.D., and One Hundred Associate Editors. Published by The F. A. Davis Company of Philadelphia.

This is Volume III. of this excellent work. Like its predecessors it is most excellent in every detail. It goes from *Dislocations* to *Infantile Myxœdema.* There are several articles of unusual excellence, besides a review at the same time of the current literature. Among these may be mentioned "Dysentery," by Dr. Flexner of Baltimore; "Endometritis," by Professor Byford of Chicago; "Dislocations and Fractures," by Professor Stimson and Dr. Keyes, Jr., of New York; "Gout," by Dr. Levison of Copenhagen; "Hysteria" and "Hypnotism," by Professor Eskridge of Denver.

There are some very handsome chromo-lithographs found, and the style of the binding is very attractive. There is much in the new arrangement of this work to be commended, and yet if we spoke our own feelings in the matter there are many points of superiority to be found in the old arrangement. As a reference work to find out what had taken place in regard to any particular subject, the old work was far more voluminous. Then, again, it placed in such a concise manner chemical reports on one particular subject which had been published during the year by men all over the world. Yet in this present arrangement each subject is treated more like that found in various text-books. It makes a valuable addition to any physician's library.

PROGRESSIVE MEDICINE. A Quarterly Digest of Advances, Discoveries and Improvements in the Medical and Surgical Sciences. Edited by Hobart Amory Hare, M.D., of Philadelphia. Volume I. Published by Lee Brothers & Co., Philadelphia and New York.

This is a new work started by Messrs. Lee Brothers, under the editorial management of one eminently fitted for the work—Dr. Hare, of Philadelphia. If we may express our opinion from a review of the present and first volume, we would say that it is a great acquisition to medical literature. This volume treats of: Surgery of the Head, Neck and Chest, Diseases of Children, Pathology, Infectious Diseases, including Croupous Pneumonia, Larnygology and Rhinology, Otology. There are several works of this character gotten out each year, but in none are we more pleased with the arrangement or more entertained in the manner in which the various subjects are presented than in this book of "Progressive Medicine." The contributors in each department are men of well-known ability, and when their own views are added to the references made it adds much to the scientific interest. It is pleasant sometimes to have the wheat and the chaff separated for you, and to have in your possession a book which will ever prove a ready reference guide as to medical advances. We predict success for this work.

---

CHEMISTRY: GENERAL, MEDICAL AND PHARMACEUTICAL, INCLUDING THE CHEMISTRY OF THE U. S. PHARMACOPŒIA. By John Attfield, F.R.S. Published by Lee Brothers & Co.

This well-known manual of chemistry by Professor Attfield has now reached its sixteenth edition, which eminently attests the extreme popularity of the book. It is a manual, clear and concise, putting in a scientific and practical manner those things which a student of chemistry should know. It is thoroughly up-to-date, and the illustrations, while few, are such as are most needed. This work is too well known for any extensive criticism.

